# One ring to rule: Cytokinesis in model and pathogenic fungi

**DOI:** 10.1371/journal.ppat.1014731

**Published:** 2026-11-03

**Authors:** Vikas Yadav, Joseph Heitman, Caroline Laplante

**Affiliations:** 1 Department of Microbiology and Immunology, University of Minnesota Medical School, Minneapolis, Minnesota, United States of America; 2 Department of Molecular Genetics and Microbiology, Duke University Medical Center, Durham, North Carolina, United States of America; 3 Department of Molecular Biomedical Sciences, College of Veterinary Medicine, North Carolina State University, Raleigh, North Carolina, United States of America; University of Wisconsin Medical School, UNITED STATES OF AMERICA

Cytokinesis, the physical separation of a mother cell into two daughter cells, is the final step of cell division and must be coordinated with nuclear division. In the eukaryotic lineage that includes amoebae, fungi, and animals (but not plants or most other eukaryotes), this step relies on a conserved actomyosin contractile ring (CR) of actin filaments and myosin-II motors [1,2]. Fungi add a requirement absent in amoebae and animal cells: they couple ring constriction and membrane ingression to the synthesis of a cell wall, or septum [1,3]. The core machinery of cytokinesis is conserved, yet fungi deploy it across strikingly different architectures: the asymmetric, site-specific divisions of budding yeasts (*Saccharomyces cerevisiae*; the pathogen *Cryptococcus neoformans*) and the symmetric, medial divisions of fission yeasts (*Schizosaccharomyces pombe*) and filamentous fungi (*Aspergillus fumigatus*, *Magnaporthe oryzae*), where septation can be uncoupled from cell separation to build hyphae (Fig 1).

**Fig 1:**
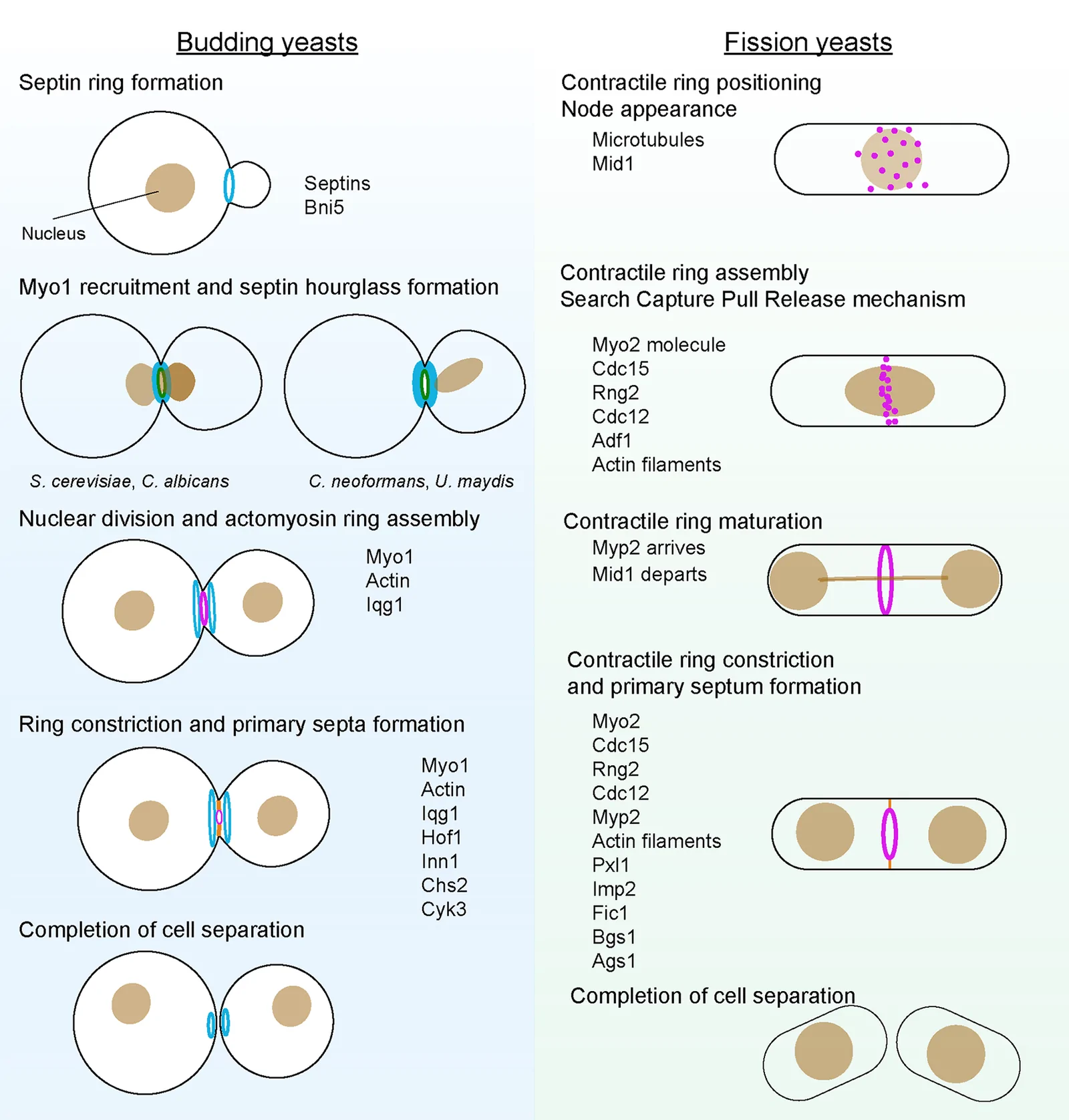
Cytokinesis in fungi. Cartoon representation showing two broadly conserved models of cytokinesis observed in the budding yeasts (left panel) and fission yeasts, as well as filamentous fungi (right panel). Key proteins associated with each step are listed for each step with protein names based on the model yeasts (*S. cerevisiae* and *S. pombe*) nomenclatures. Myo1 is the budding-yeast myosin-II, whereas Myo2 and Myp2 are the fission-yeast myosin-II paralogs. Conserved identities of the proteins labeled by gene name are: Bni5 (septin linker), Iqg1 and Rng2 (IQGAP), Hof1 and Cdc15 (F-BAR), Mid1 (anillin), Cdc12 (formin), Inn1 (C2 domain), Chs2 (chitin synthase), Cyk3 (transglutaminase-like), Adf1 (cofilin), Pxl1 (paxillin), and Imp2 and Fic1 (F-BAR/C2 domain). In the diagrams, cyan denotes septins, green the early myosin ring, magenta the mature actomyosin (contractile) ring that forms after recruitment of actin and accessory proteins, and tan the nucleus.

Whether proteins involved in cytokinesis hold special relevance for fungal pathogenesis and provide viable treatment candidates is not obvious, apart from their essential role in cell proliferation. The machinery is broadly conserved, and the parts list in a pathogen resembles that of its non-pathogenic relatives more than that of other pathogens. We argue that the interesting biology lies not only in a pathogen-specific parts list but also in how an essential, conserved process is re-regulated and re-deployed under host conditions. Here, we compare cytokinesis and septation across human and plant fungal pathogens, ask where their regulation and morphology diverge from the model yeasts, and consider why these divergences make the machinery an attractive source of antifungal targets. This comparison identifies host-adapted regulation, rather than novel components, as the recurring theme of cytokinesis in pathogenic fungi, with the core machinery being largely conserved among various species.

## Position, assembly, and constriction of the actomyosin ring in fungi

Septins are a conserved family of GTP-binding proteins that assemble into hetero-oligomeric complexes and polymerize into filaments and higher-order structures, including the rings that mark fungal division sites [4,5]. In budding yeast, the Rho-family GTPase Cdc42 and its effectors recruit septins to the incipient bud site [4,5]. Budding yeasts employ a ring of septins to position and anchor the CR at the bud site throughout cytokinesis. This septin ring is highly dynamic; it acts as a scaffold capable of adopting different architectural shapes and polymeric conformations to stabilize the CR [4,5]. The connection between the septin scaffold and the CR is mediated by specific linker proteins, such as Bni5 and the IQGAP homolog Iqg1 [6–9]. The CR itself is composed of actin filaments and myosin-II motors, supported by a structural framework of formin Bni1, Iqg1, and F-BAR domain protein Hof1 [2,10,11]. Hof1 plays a particularly critical role in anchoring the CR and maintaining its connection with the plasma membrane [12–14], and is required for proper septation in the next step [11]. As the ring constricts, it reduces in size and proteins are shed in proportion to the decreasing circumference to maintain a constant protein concentration, thereby preventing defective cytokinesis [15–17]. Notably, the motor domain of myosin-II in budding yeast, Myo1, is dispensable for cytokinesis [17].

The molecular organization of the CR is best understood in the fission yeast *S. pombe*. In this yeast, the position of the nucleus, centered within the cell by the dynamic actions of interphase microtubules, determines the site of assembly of the CR [18–20]. Over 150 cytokinetic proteins have been identified in *S. pombe*, many of which are conserved from yeast to animal cells [21]. Membrane-bound protein complexes called nodes regulate and build the contractile ring in *S. pombe* starting in interphase. Two types of interphase nodes assemble at the cell membrane during interphase and persist into mitosis, where they contribute to cytokinetic node formation [22,23]. The Cdr2 kinase organizes the type 1 interphase node proteins, which include the anillin-like Mid1 and the kinases Cdr1 and Wee1, while the Type 2 nodes contain Blt1, the Rho GEF Gef2, and the kinesin-like Klp8. Cytokinetic nodes contain the F-BAR protein Cdc15, the formin Cdc12, the IQGAP homolog Rng2, the anillin homolog Mid1, and the myosin-II Myo2 molecule composed of the Myo2 heavy chain, essential light chain Cdc4, and regulatory light chain Rlc1 [24,25]. Nodes coalesce into the CR via the search, capture, pull, and release (SCPR) mechanism [26]. Through this mechanism, Myo2 molecules capture actin filaments polymerized by Cdc12 in neighboring nodes and then pull on the actin filaments, bringing the connected nodes together. Connections between nodes must be transient by the action of the actin filament-severing protein Cofilin/Adf1 to ensure the formation of a continuous ring without clumps of nodes [26,27]. Super-resolution in live *S. pombe* cells revealed the molecular organization of proteins within nodes and that nodes remain in the CR during constriction [28,29]. Nodes likely function in the ring to connect the actin filament bundle and thus transmit contractile forces to the plasma membrane and possibly to the cell wall [28–31]. A second F-BAR protein, Imp2, also participates in anchoring the CR to the division site, but it remains unknown how it connects to the actin bundle [18,32–36].

In *S. pombe*, cytokinetic proteins distribute into functional layers within the CR with the layer closest to the plasma membrane likely acting as an anchoring layer that transmits the contractile forces from the inner actin filament bundle layer to the plasma membrane [37,38]. The two myosin-II, Myo2 and Myp2, cooperate to assemble and constrict the CR. They distribute between these two layers, with Myo2 in the nodes connected to the plasma membrane and Myp2 in the inner layer. A myosin-V, Myo51, localizes with Myp2 in the inner layer of the ring and supports the function of Myp2 and Myo2 during cytokinesis, but its exact function remains unclear [37,39]. Although the bulk of the actin filament bundle lies in the inner layer, myosin motors distribute across the outer and inner layers, suggesting that contractile forces likely arise in both layers.

## Actomyosin ring in pathogenic fungi

Among the fungal pathogens, septins that act as anchors for CR assembly are the most extensively studied group of proteins, whereas studies on components of CR itself are limited. For example, in the human pathogen *Candida albicans,* a human gut commensal, septins are localized to the division site to form ring structures and are essential for morphogenesis, a key virulence factor [40–42]. Among CR-specific components, myosin-II, Iqg1, and formins have been described with conserved roles in cytokinesis [7,43]. Interestingly, some components have evolved distinct functions in this species; for example, Hof1 orthologs function in DNA damage responses rather than purely in cytokinesis [44]. Similar to *C. albicans*, septins are required for the morphological transition in the opportunistic pathogenic budding yeast *C. neoformans* and also play a role in nuclear dynamics [45].

The corn smut plant pathogen *Ustilago maydis* grows as a budding yeast and undergoes a morphological transition to hyphal growth during infection. Septins affect this morphological transition as well as polarity in growing yeast cells [46,47]. The myosin-II essential light chain Cdc4 also localizes to the site of division along with the septins and differs from Hof1/Cdc15 localization, which localizes onto the CR at a later stage. Deletion of Hof1/Cdc15 causes defective cell separation, nuclear distribution, and also affects septin ring assembly specifically [48]. Localization of Hof1/Cdc15 depends on the formin Drf1, which is necessary for CR assembly and septation during hyphal growth and infection [49]. Similar to their roles in *U. maydis*, septins are critical for growth and stress responses, but they also play specialized roles in hyphal branching and the formation of appressoria (infection structures) in species like *M. oryzae*, the pathogen responsible for causing rice blast disease [50–53]. In this organism, Hof1/Cdc15 orthologs and myosin-II homologs are crucial for hyphal growth and conidiation, the process of asexual reproduction in filamentous fungi [54,55]. These functions of septins and myosin II homologs are similarly conserved in the human fungal pathogen *A. fumigatus*, where they play key roles in septation and conidiation [56,57].

## Septation in pathogenic fungi

The CR constriction in fungi occurs concurrently with the deposition of a primary septum. In budding yeast, the specialized Class II chitin synthase polymerizes the chitin of the primary septum, a process orchestrated by the Ingression Progression Complex (IPC), which includes the myosin-II Myo1, the IQGAP homolog Iqg1, Cdc15 homolog Hof1, the C2-domain protein Inn1, the chitin synthase Chs2, and the transglutaminase-like protein Cyk3 [11,58–62]. Proper assembly of the IPC activates Chs2, driving primary septum formation. This includes direct interactions of the Inn1 C2 domain, and of Cyk3, with the catalytic domain of Chs2, resulting in its activation [59]. During this stage, septins split into a double ring structure on both sides of the primary septum to recruit proteins for secondary septum formation. Finally, the primary septum is dissolved to separate the daughter cells [11]. In the fission yeast *S. pombe*, chitin is absent from the vegetative cell wall [63,64]. While chitin is a standard structural component in most other fungi, *S. pombe* has evolved to use alpha- and beta-1,3-glucan produced by Ags1 and Bgs1 instead to provide the mechanical strength usually conferred by chitin [64,65]. In *S. pombe*, Chs2 is a functional protein that localizes to the growing edge of the septum yet lacks chitin synthase activity [66], acting instead as a regulator of the septation machinery and cell separation [63,67]. However, the *S. pombe* model does not extend fully to filamentous pathogens: unlike *S. pombe*, moulds such as *A. fumigatus* deposit chitin in their septa, so the fission-yeast paradigm captures their CR dynamics but not the composition of their septum [68,69].

In *C. albicans*, the class II chitin synthase, Chs1, is essential for cell survival, and the related class I chitin synthase Chs2 localizes to septa during division [70,71]. The cryo-EM structures of *C. albicans* Chs1 and Chs2 were recently solved and provide key insights into their regulation, as well as identify key domains that can be explored for drug development [72,73]. In *Cryptococcus deneoformans*, the IPC components Imp2, Cts1, and Chs6 (Cdc15/Imp2, Inn1, and Chs2 orthologs, respectively) were recently described and are structurally conserved but uniquely regulated [11,74]. A genetic screen revealed that their function is governed by the calcium-calcineurin signaling cascade via dephosphorylation of Imp2 [74]. Calcineurin is essential for C. *deneoformans* growth at 37 °C, and dominant mutations in any of these three components bypass the requirement for calcineurin for growth during temperature stress at 37 °C. This suggests that while the structural machinery resembles that of model budding yeasts, the regulatory wiring has diverged to support survival in the host at higher temperatures.

Observations in *S. pombe* provide an important context for understanding these unique regulatory mechanisms in *C. deneoformans*. In *S. pombe*, calcineurin regulates the function of the F-BAR protein Cdc15 through the paxillin-like protein Pxl1 [75]. This pathway highlights a regulatory conservation shared between *S. pombe* and *Cryptococcus* species, which harbor three paxillin orthologs. Consequently, the cytokinesis machinery in *Cryptococcus* species appears to be a hybrid system, harboring features shared with both *S. cerevisiae* and *S. pombe*.

## Role of the cytokinesis machinery in fungal pathogenesis

A caveat on terminology is warranted. Many proteins discussed here are not dedicated to cytokinesis: septins, actin regulators, and F-BAR proteins also govern polarity, morphogenesis, stress responses, and, for *C. albicans* Hof1, the DNA-damage response. We therefore utilize “cytokinesis machinery” for the core CR and septation apparatus while recognizing that individual components are pleiotropic, and we are explicit when a link to pathogenesis runs through a non-cytokinetic function rather than through cytokinesis itself.

While studies in pathogenic fungi focusing on the cytokinesis machinery have been sparse, those limited studies have found these proteins to be critical for the pathogenicity of these organisms. Septins, the most studied component of this machinery, are required for virulence in some pathogenic fungi but dispensable in others [42,45–47,50,52,57]. Many of the components of the CR have been described in *C. albicans* with direct implications for virulence through regulation of cell growth, polarity, and morphological transition [7,41,43]. Characterization of IPC components in *C. deneoformans* also revealed a requirement of these proteins for growth at 37 °C, a defined virulence attribute in this species [74]. Formation of the infection structure appressoria in *M. oryzae* is essential for its virulence, a process that is directly regulated by Cdc15 homologs [54]. Together, these studies suggest that CR components and proteins required for septation play vital roles during fungal infections in both animals and plants.

While most of the cytokinesis machinery and its core components are highly conserved across eukaryotes, including humans, the synthesis of a septum is a major difference between animal and fungal cells. Furthermore, several key accessory proteins involved in this process, such as C2-domain-containing protein Inn1, are highly divergent even among different fungal species. These features make the cytokinesis and septation machinery appealing targets for drug development purposes. Septum synthesis has no counterpart in animal cells, so the enzymes that build it are intrinsically selective targets. The precedent is established: echinocandins, a frontline antifungal class, act by inhibiting the β-1,3-glucan synthase Fks1, a cell-wall biosynthetic enzyme tied to septation [76]. Chitin synthases offer a parallel opportunity: nikkomycin Z inhibits chitin synthases [77,78], and a Chs1-specific inhibitor arrests growth of *C. albicans* [72,79]. Because the regulation of the machinery is rewired in pathogens, as in the calcineurin control of the ingression progression complex in *Cryptococcus*, the regulators themselves may also be exploitable, particularly where they are required for growth at host temperature. Systematic comparison of these proteins across pathogens is therefore likely to reveal selective, and in some cases species- or morphology-specific, antifungal targets.

## Prospects and further steps

Cytokinesis is generally associated with cell proliferation. However, proteins involved in cytokinesis are also responsible for defining cell shape and size, an important characteristic of fungi that is crucial for their virulence. The morphological transition of *C. albicans* from yeast to hyphae is essential for its virulence. Similarly, *C. neoformans* undergoes several morphological transitions during infection (titan cells, seed cells, ovoid cells), each of which likely requires distinct cytokinetic regulation. *M. oryzae* infection involves formation of an appressorium during which not only the cell shape but even the septation is differentially regulated. Despite such a critical role in cell proliferation and pathogenesis, the cytokinesis machinery in pathogenic fungi remains largely uncharacterized in terms of its structure, dynamics, and regulation. As described here, current studies have only identified the core, most conserved components of this machinery, with limited efforts to understand the assembly of this large, highly dynamic macromolecular set of protein complexes (Table 1). Our functional understanding of cytokinesis in pathogenic fungi rests largely on extrapolation from the model yeasts, *S. cerevisiae* for the pathogenic yeasts and *S. pombe* for the filamentous fungi. These extrapolations, however, remain largely untested: few studies have directly confirmed that the cytokinetic mechanisms defined in the model yeasts operate the same way in the pathogens. Additionally, there are substantial differences between these two groups (budding yeasts and fission yeasts) that need to be explored further. For example, *C. deneoformans* shows structural conservation of a complex present in *S. cerevisiae,* and regulatory conservation similar to *S. pombe,* suggesting a rewiring of this complex throughout fungi [11,60,74,75]. *C. albicans,* Hof1 plays a role in the DNA damage response [44], suggesting that these proteins are required for additional roles in these pathogens. Furthermore, pathogenic fungi exhibit diverse cell shapes, sizes, and morphologies and might require specific accessory proteins that work with core components of cytokinesis machinery. As a result, the differential regulation, function, and evolution of cytokinesis machinery during these morphological transitions cannot be described based on our knowledge in model yeasts and requires more specific, focused approaches in pathogenic fungi.

**Table 1:** Homologs/Orthologs of key proteins involved in cytokinesis. The name descriptions listed in the first column are common names, and species-specific ORF numbers, with their names in parentheses where known, are listed for each case. Multiple Chs2 homologs/orthologs are present in most species, and those are listed as such.

Protein family	*Saccharomyces cerevisiae*	*Schizosaccharomyces pombe*	*Candida albicans*	*Cryptococcus deneoformans*	*Ustilago maydis*	*Magnaporthe oryzae*	*Aspergillus fumigatus*
Anillin	YJR092W (Bud4)	SPCC4B3.15 (Mid1)				MGG_12956	
Myosin-II	YHR023W (Myo1)	SPBC146.13c (Myo2) SPAC4A8.05c (Myp2)	C5_01650C_A	CNC06470	UMAG_03286	MGG_03060	Afu5g08700
IQGAP homolog	YPL242C (Iqg1)	SPAC4F8.13c (Rng2)	C7_01840W_A	CNB02580	UMAG_10730	MGG_02076 MGG_03700	Afu2g03640
F-BAR domain containing proteins	YMR032W (Hof1)	SPAC20G8.05c (Cdc15) SPBC11C11.02 (Imp2)	C4_00370W_A	CNB00620 (Imp2)	UMAG_00168	MGG_11899 (MoCdc15)	Afu3g10250
C2 domain containing protein	YNL152W (Inn1)	SPBC83.18c (Fic1)	CR_02740W_A	CNG01630 (Cts1)	UMAG_06398	MGG_01150	Afu2g01790
Chitin synthases	YBR038W (Chs2)	SPBC1709.01 (Chs2) SPAC13G6.12c (Chs1)	CR_09020C_A (Chs1)	CNN02110 (Chs6) CNC00050 (Chs8)	UMAG_04290 (Chs2) UMAG_10120 (Chs3) UMAG_10117 (Chs4)	MGG_01802 (Chs1) MGG_04145 (Chs2) MGG_09551 (Chs3)	Afu2g01870 (ChsA) Afu4g04180 (ChsB)
Cytokinesis-associated Papain-like cysteine peptidase	YDL117W (Cyk3)	SPAC9G1.06c (Cyk3)	C1_06700W_A			MGG_12117	Afu5g03400
Paxillin-like homolog	YKR090W (Pxl1)	SPBC4F6.12 (Pxl1)	C6_02090C_A	CNB03950 CNH01575 CNH04580	UMAG_01663 UMAG_06412 UMAG_01834	MGG_06198 MGG_05738	Afu2g01000 Afu4g12270
Actin	YFL039C (Act1)	SPBC32H8.12c (Act1)	C1_13700W_A (Act1)	CNA04650 (Act1)	UMAG_11232	MGG_03982	Afu6g04740 (Act1)
α-actinin		SPAC15A10.08 (Ain1)		CNC06000	UMAG_04312	MGG_06475	Afu5g08300
Actin capping proteins	YKL007W (Cap1) YIL034C (Cap2)	SPAC12B10.07 (Acp1) SPAC631.01c (Acp2)	CR_01180W_A (Cap01) C4_01950W_A (Cap2)	CNB04560 (Cap1) CNB03880 (Cap2)	UMAG_00423 UMAG_11177	MGG_12818 (CapB) MGG_09902 (CapA)	Afu6g10060 (Cap1) Afu1g03060 (Cap2)
Tropomyosin	YNL079C (Tpm1) YIL138C (Tpm2)	SPAC27F1.02c (Cdc8)	CR_08460W_A (Tpm2)	CNF02720 (Tpm1)	UMAG_11985	MGG_08078	Afu7g04210
Formins	YNL271C (Bni1) YIL159W (Bnr1)	SPAC1F5.04c (Cdc12) SPCC895.05 (For3)	C1_12960C_A (Bni1) CR_00070W_A (Bnr1)	CNJ02400 (Bni1)	UMAG_01141 UMAG_12254	MGG_17697	Afu6g04940
Cofilin	YLL050C (Cof1)	SPAC20G4.06c (Adf1)	C5_00370W_A (Cof1)	CNC05990 (Cof1)	UMAG_04314 (Cof1)	MGG_03164	Afu5g10570

We think that the cytokinesis machinery should be considered an important process required for virulence, as it affects cell proliferation and shape, both of which are essential for virulence. The findings of pleiotropic roles for these proteins in pathogenic fungi make them even more crucial to understand, especially given that the pleiotropic roles are sometimes in key cellular processes such as DNA damage repair. Furthermore, understanding the cytokinesis machinery in these organisms has the potential to identify species-specific or even morphology-specific proteins in pathogenic fungi that are absent in humans and/or plants.
